# VOICE FIRST TECHNOLOGY: SIMPLIFYING LIFE FOR COMMUNITY-DWELLING OLDER ADULTS LIVING WITH DEMENTIA

**DOI:** 10.1093/geroni/igz038.1432

**Published:** 2019-11-08

**Authors:** Debra J Sheets, Marilyn Malone, Stuart W MacDonald, Carl Asche, Andre Smith

**Affiliations:** 1 University of Victoria, Victoria, British Columbia, Canada; 2 Island Health, Victoria, British Columbia, Canada; 3 University of Illinois-Peoria, Peoria, Illinois, United States

## Abstract

Voice first technology offers older adults with dementia support that may maintain independence, reduce social isolation and improve quality of life (QoL). This study investigates the impact of a voice-controlled technology customized to the needs of participants living with dementia and their caregivers. A mixed methods design focused on psychosocial factors and usability characteristics. The purposive sample consisted of older adults with dementia (n=12) and their care partners (n=12)) living independently in the community. Validated measures for cognition, depression, caregiver burden, quality of life and usability were included. Qualitative in-home interviews were conducted to assess impact on social connections and independence. Results indicate that voice first technology can reduce caregiver burden and can support the independence and QoL of older adults with dementia. The discussion considers the value of low cost voice first technology as a way to support older adults with dementia and their caregivers.

